# Supplementary figures and images for: Can ChatGPT assist authors with abstract writing in medical journals? Evaluating the quality of scientific abstracts generated by ChatGPT and original abstracts
Source: PLoS One. 2024 Feb 14;19(2):e0297701. doi: 10.1371/journal.pone.0297701 (PMC10866463; doi:10.1371/journal.pone.0297701)

# **S1 Fig: Prompt for Abstract Generation and ChatGPT Response**

**
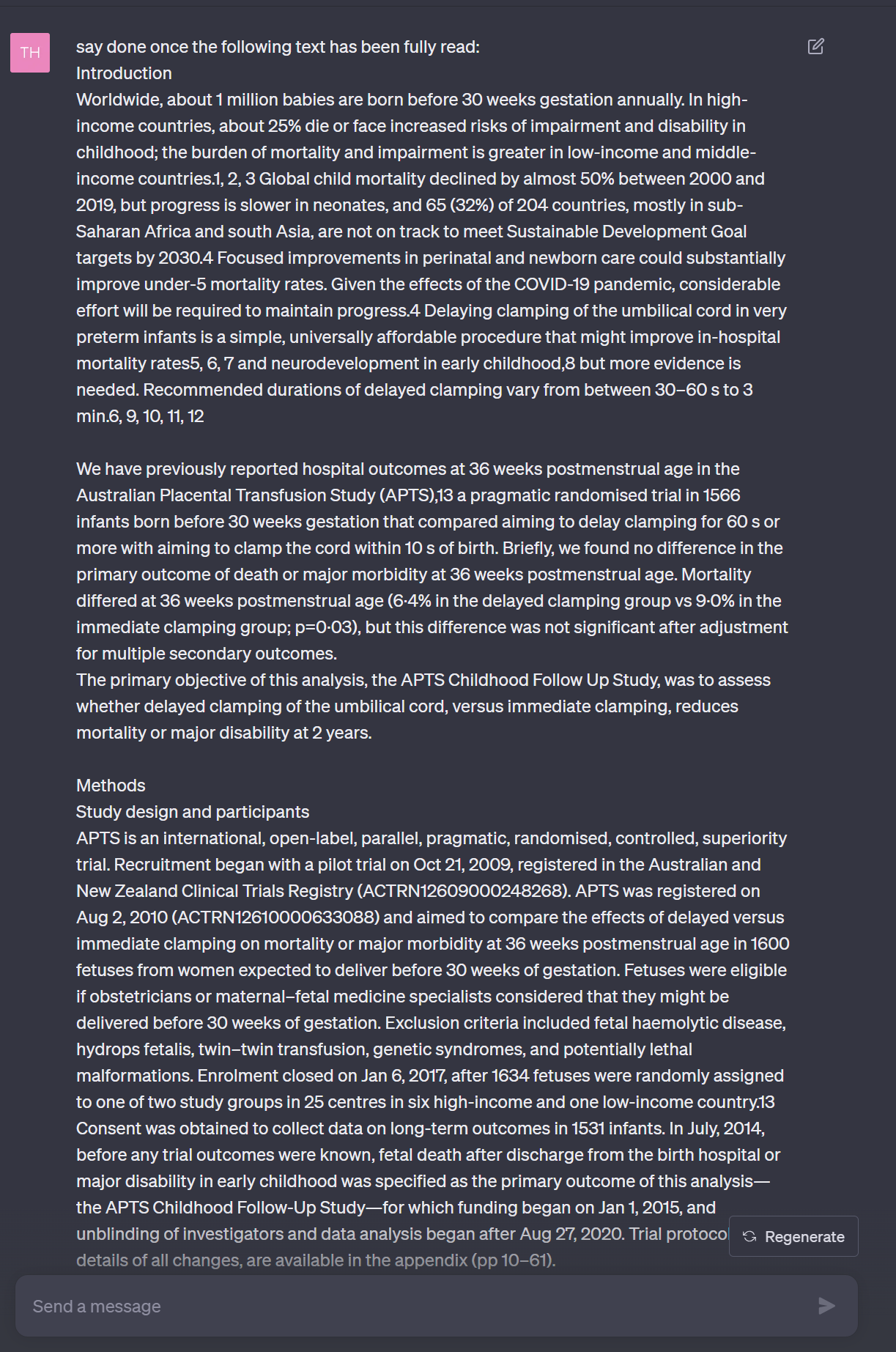
**


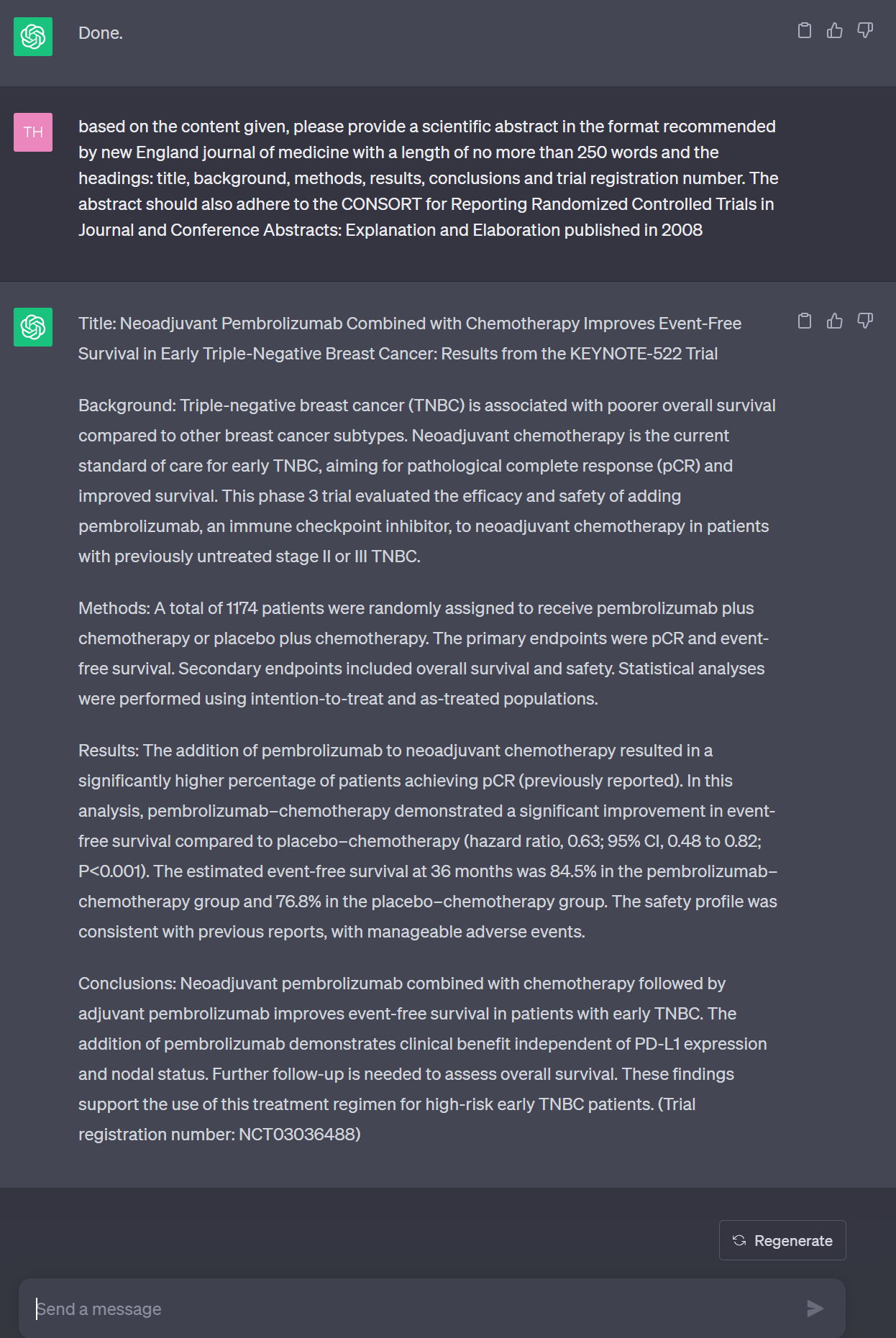

Supplement: S1 Fig — (DOCX) [file pone.0297701.s001.docx]
