# Supplementary material for: Can ChatGPT assist authors with abstract writing in medical journals? Evaluating the quality of scientific abstracts generated by ChatGPT and original abstracts
Source: PLoS One. 2024 Feb 14;19(2):e0297701. doi: 10.1371/journal.pone.0297701 (PMC10866463; doi:10.1371/journal.pone.0297701)
Supplement: S2 Fig — (DOCX) [file pone.0297701.s002.docx]

# **S2 Fig: CONSORT-A Checklist**

**Title**

1. **Title**: do the authors state that the participants were randomly allocated to their respective groups?

**Trial Design**

1. **Trial design**: do the authors state the type of randomized controlled trial performed? Have they included details such as parallel, cluster, or non-inferiority? Just stating that it is a randomized controlled trial would not be sufficient.

**Methods**

1. **Participants:** do the authors state the eligibility criteria for participants? Can the reader assess the generalizability of the trial and determine the applicability of the study’s results to their own setting?
2. **Participants:** is there a clear description of the setting (level of care provided at the study site) in which the participants were studied? For example, study was carried out in primary/secondary/tertiary care centre, health clinic, or community clinic. This does not have to mention geographic setting. If it includes “university” this implies the centres is tertiary and therefore scores 1. Number of sites AND the level of care must be included to score.
3. **Interventions**: interventions given to each group. Important features, such as dose, route of administration, duration, and the details to the surgical procedure must be included.
4. **Objective**: Is there a clear statement of the specific objective or hypothesis addressed in the trial?
5. **Outcome**: do the authors clearly define the primary outcome measure of the study?
6. **Randomisation**: do the authors explain the method for random assignment of participants? Are we told how random sequence was generated?
7. **Randomisation:** do the authors describe the method of allocation concealment?
8. **Blinding**: is the study described as “blinded or “masked” AND state who was blinded> Double blind means both the assessors and the participants were blinded and hence scores a point.

**Results**

1. **Numbers randomised**: do the authors mention the number of participants randomised to each group?
2. **Numbers analysed**: is the number of participants analysed in each group mentioned?
3. **Outcome**: is the primary outcome result stated for EACH group?
4. **Outcome**: Is the estimated effect size and its precision mentioned. For binary outcomes, the effect size could be the relative risk, relative risk reduction, odds ratio, or risk difference. For survival time data, the measurement could be the hazard ratio or difference in median survival time. For continuous data, the effect measure is usually the difference in means. Confidence interval should be included for precision of the results data.
5. **Harms**: are the important adverse effects or side effects mentioned for EACH group? This does not require effect size or confidence interval however numbers should be included to illustrate this. If no important adverse events have occurred, this should be stated explicitly.

**Conclusions**

1. **Conclusion**: do the authors draw a general interpretation of the results?

**Registration**

1. **Trial registration**: is the registration number included?

**Funding**

1. **Funding**: is the source of funding stated?
