# Supplementary material for: Can ChatGPT assist authors with abstract writing in medical journals? Evaluating the quality of scientific abstracts generated by ChatGPT and original abstracts
Source: PLoS One. 2024 Feb 14;19(2):e0297701. doi: 10.1371/journal.pone.0297701 (PMC10866463; doi:10.1371/journal.pone.0297701)
Supplement: S1 Table — (DOCX) [file pone.0297701.s003.docx]

# **S3 Table: Comparison of the adherence of CONSORT-A checklist items by the original abstracts vs GPT 3.5 vs GPT 4-generated abstracts**

| **Criterion assessed** | **Original abstract (N=62), (%)** | **GPT 3.5-generated (N=62), %** | **GPT 4-generated (N=62), %** | **Original vs GPT 3.5 P-values** | **Original VS GPT 4 P-values** | **GPT 3.5 vs GPT 4 P-values** |  |
| --- | --- | --- | --- | --- | --- | --- | --- |
| 1. Title | 42 (67.74%) | 42 (67.74%) | 37 (59.68%) | 1.000 | 0.455 | 0.455 |  |
|  |  |  |  |  |  |  |  |
| 2. Trial design | 26 (41.94%) | 18 (29.03%) | 13 (20.97%) | 0.189 | **0.020** | 0.407 |  |
| **METHODOLOGY** |  |  |  |  |  |  |  |
| 3. Participants, eligibility criteria (a) | 47 (75.81%) | 34 (54.84%) | 11 (17.74%) | **0.024** | **<0.001** | **<0.001** |  |
| 4. Participants, description of study setting (b) | 23 (37.10%) | 16 (25.81%) | 5 (8.06%) | 0.246 | **<0.001** | **0.017** |  |
| 5. Interventions | 53 (85.48%) | 36 (58.06%) | 19 (30.65%) | **0.001** | **<0.001** | **0.004** |  |
| 6. Objective | 43 (69.35%) | 56 (90.32%) | 37 (59.68%) | **0.007** | 0.348 | **<0.001** |  |
| 7. Primary outcome | 60 (96.77%) | 53 (85.48%) | 35 (56.45%) | 0.058 | **<0.001** | **0.001** |  |
| 8. Randomisation, method (a) | 10 (16.13%) | 1 (1.61%) | 0 (0.00%) | **0.012** | **0.003** | 1.000 |  |
| 9. Randomisation, allocation concealment (b) | 3 (4.84%) | 0 (0.00%) | 0 (0.00%) | 0.242 | 0.242 | NA |  |
| 10. Blinding | 37 (59.68%) | 33 (53.23%) | 20 (32.26%) | 0.587 | **0.004** | **0.029** |  |
| **RESULTS** |  |  |  |  |  |  |  |
| 11. Numbers randomised | 51 (82.26%) | 19 (30.65%) | 11 (17.74%) | **<0.001** | **<0.001** | 0.142 |  |
| 12. Numbers analysed | 32 (51.61%) | 2 (3.23%) | 2 (3.23%) | **<0.001** | **<0.001** | 1.000 |  |
| 13. Outcome, results (a) | 59 (95.16%) | 28 (45.16%) | 12 (19.35%) | **<0.001** | **<0.001** | **0.004** |  |
| 14. Outcome, effects size and precision (b) | 58 (93.55%) | 26 (41.94%) | 10 (16.13%) | **<0.001** | **<0.001** | **0.003** |  |
| 15. Harms | 41 (66.13%) | 9 (14.52%) | 8 (12.90%) | **<0.001** | **<0.001** | 1.000 |  |
| 16. Conclusions | 61 (98.39%) | 62 (100.00%) | 58 (93.55%) | 1.000 | 0.361 | 0.127 |  |
| 17. Trial registration | 55 (88.71) | 37 (59.68%) | 26 (41.94%) | **<0.001** | **<0.001** | 0.072 |  |
| 18. Funding | 36 (58.06%) | 19 (30.65%) | 18 (29.03%) | **0.004** | **0.002** | 1.000 |  |
| *Pearson’s χ2 test* *was used to compare the performance of each abstract subgroup.*  Significant p-values (P<0.05) have been **emboldened.** | | | | | | |  |
