# Supplementary material for: Can ChatGPT assist authors with abstract writing in medical journals? Evaluating the quality of scientific abstracts generated by ChatGPT and original abstracts
Source: PLoS One. 2024 Feb 14;19(2):e0297701. doi: 10.1371/journal.pone.0297701 (PMC10866463; doi:10.1371/journal.pone.0297701)
Supplement: Attachment — Submitted filename: Respponse to Reviewers.docx [file pone.0297701.s004.docx]

Dear Editor,

We would like to thank the editor and reviewers for their detailed feedback on our manuscript. We have addressed all comments raised by the reviewers and responded with detailed point-by-point explanations. Marked-up copy of the manuscript highlights the changes made in response to the reviewer’s comments, and all page and line numbers mentioned below refer to the marked-up copy. We hope that the changes made to our manuscript adequately address the reviewers’ comments and hence allow for the publication of our novel study.

Yours sincerely,

Taesoon Hwang

**Reviewer’s Comments**

**Reviewer #1: This is an interesting study. you may modify the flow chart as the current one is more vertically oriented. To be specific, PubMed and Medline are two different terms, one is a search engine and one is a bibliographic database**

We thank the reviewer for their comment on the figure. We have edited the presentation of the flowchart in Figure 1 and have clarified that Medline search was conducted through the PubMed platform in Line 96.

**Reviewer #2: With a recent surge of application of language learning models in clinical medicine and dentistry, I find this study timely and relevant. The study is methodologically sounds, however, I have few minor suggestions that I have highlighted below:**

**1. Line 1: Since thee topic is focussed on medical journals, I suggest to modify the title to "Can ChatGPT Assist Authors with Abstract Writing in Medical Journals? Evaluating the Quality of Scientific Abstracts Generated by ChatGPT and Original Abstracts"**

We appreciate the reviewer’s suggestion to modify the title of our submitted work. We agree that specifying “Can ChatGPT Assist Authors with Abstract Writing in Medical Journals?” better captures the essence of our work and clarifies the context in which the abstracts were evaluated.

**2. Line 79: Rephrase the line. Equator is a database and it did not introduce CONSORT-A. It is an independent research group.**

We thank the reviewer for the correction. As suggested, we have addressed this point in Lines 77-86.

**3. Line 95: Elaborate on how journals were selected?**

We appreciate the reviewer’s comment on elaborating the journal selection process. We agree with this point and have included a detailed explanation in Lines 96-114.

**4. Line 130: Explain the term "hallucination"**

We thank the reviewer for this point and appreciate that “hallucination” can be an unfamiliar term for new readers. We have clarified the definition of hallucination in Lines 149-152.

**5. Line 143: I see the authors are trained and calibrated. Include reliability scores for the examiners**

We appreciate the reviewer’s point and recognise the need to demonstrate reliability in the assessment of abstracts between our evaluators. We have included an additional subsection highlighting a substantial agreement between our evaluators in Lines 278-281 and we have included a table to illustrate this (see Table 6).

**6. Table 1: Although this topic is specific to general journals, In addition to general medicine and surgery, I see more emphasis on three specialties: pediatrics, O&G, and Psychiatry. Kindly explain the reason for this focus.**

We thank the reviewer for this comment. In this study, our overarching goal was to assess ChatGPT’s capabilities across a broad spectrum of medical specialties. While general medicine and surgery are core areas of focus, we also recognised the importance of including other pivotal specialties such as paediatrics, obstetrics and gynaecology and psychiatry. These fields represent distinct and critical aspects of medical science and by incorporating them, we sought to provide a more comprehensive evaluation of ChatGPT’s utility in various medical contexts.

The table’s apparent emphasis on paediatrics, obstetrics and gynaecology, and psychiatry should not be interpreted as an additional focus on these fields. Instead, it demonstrates our deliberate effort to ensure that the study reflects the broad spectrum of medical disciplines. For ease of presentation and to improve the table’s readability, we grouped a multitude of disciplines under the umbrella terms ‘Surgery’ and ‘Medicine’. These broad categories are further delineated into nine sub-specialties each as represented by the superscripts a and b. This structuring choice was made purely for presentation purposes. It is important to clarify that every specialty whether it falls under the broad categories or is one of the three specifically mentioned fields, holds equal value to our study and this approach highlights the comprehensive nature of our analysis meanwhile it reinforces the applicability of our findings across the diverse medical fields. We have explained the rationale for including paediatrics, O&G, and psychiatry in Lines 105-114.
